# First person – Katie Barnes

**DOI:** 10.1242/bio.062661

**Published:** 2026-05-20

## Abstract

First Person is a series of interviews with the first authors of a selection of papers published in Biology Open, helping researchers promote themselves alongside their papers. Katie Barnes is first author on ‘ Isolation, validation, and long-term culture of mouse ear fibroblasts’, published in BiO. Katie is a PhD graduate student researcher in the lab of Laura Knoll at University of Wisconsin-Madison School of Medicine and Public Health, Madison, WI, USA, investigating novel and efficient model systems for research of intracellular microbes.

**Figure:**
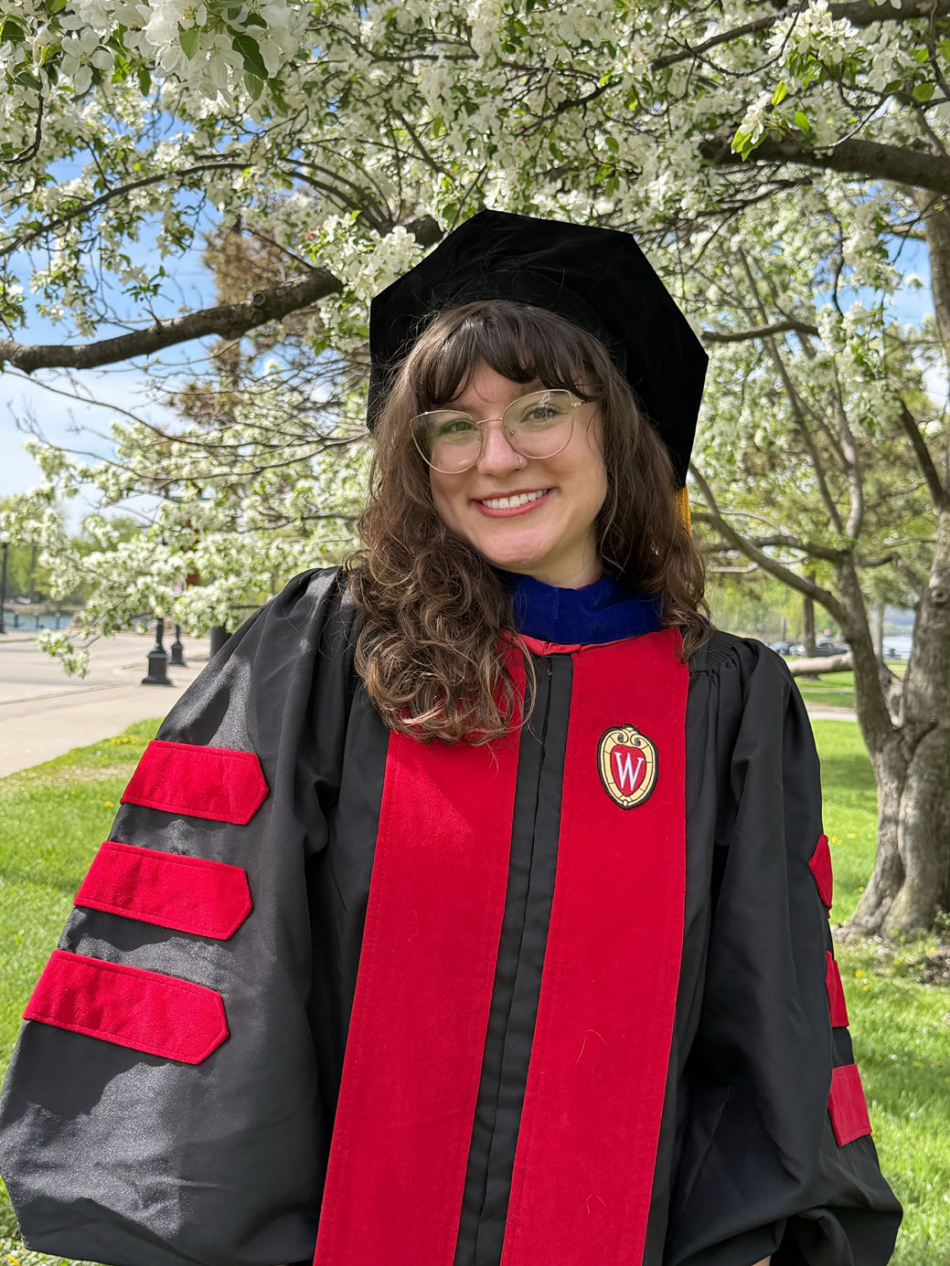
Katie Barnes

**Describe your scientific journey and your current research focus**

I have always loved puzzles. To me, science is a large, intricate puzzle, with the pieces upside down. Growing up in rural California, becoming a scientist was not something I could picture for myself. When I started college at UC, Santa Cruz, I took every opportunity to learn more about the cutting-edge of basic scientific research. Through that curiosity, I connected with a then graduate student, Dr Laura Chappell, in Dr Bill Sullivan's lab, studying the endosymbiotic relationship between the bacterium, *Wolbachia*, and the parasitic worm, *Brugia*. Drawn to the implications of basic research for human health, I worked in the Sullivan Lab for the remaining 2 years of my undergraduate degree, then stayed an additional year as an NIH PREP Scholar. I arrived at the University of Wisconsin-Madison carrying a strong interest in parasitology, human health and science advocacy, where I joined Dr Laura Knoll's lab to study host–parasite interactions between a host's arginine metabolism in *Toxoplasma gondii* infection. As I complete my PhD, I am excited to bring these skills and passions into industry diagnostics and advance science advocacy.

To me, science is a large, intricate puzzle, with the pieces upside down

**Who or what inspired you to become a scientist?**

I have always wanted to pursue a career that helps people. What that looked like evolved as my knowledge of the world grew. My high-school science teacher, a real-life Mrs Frizzle, was the first person who made me feel like science was something that I could do myself, not just admire from a distance. Every step since then, I have been fortunate to be surrounded by passionate mentors who helped me grow into the scientist I am today.

**How would you explain the main finding of your paper?**

Scientists often use cells grown in a dish to study infections, diseases and potential treatments. The conditions those cells are grown in, what they are fed or how they are handled can quietly affect the results of an experiment without researchers noticing. In this paper, we identified conditions and protocols for optimal growing of a specific skin cell type, derived from mouse ears. Consistent conditions yield more reliable and reproducible science.

In this paper, we identified conditions and protocols for optimal growing of a specific skin cell type, derived from mouse ears

**What are the potential implications of this finding for your field of research?**

Mouse ear fibroblasts are used in the Knoll Lab as a model host for the intracellular parasite, *Toxoplasma gondii*. The findings of our research are further evidence of how important controlled media conditions are for cell culture. As our lab likes to say, media matter!

**Figure:**
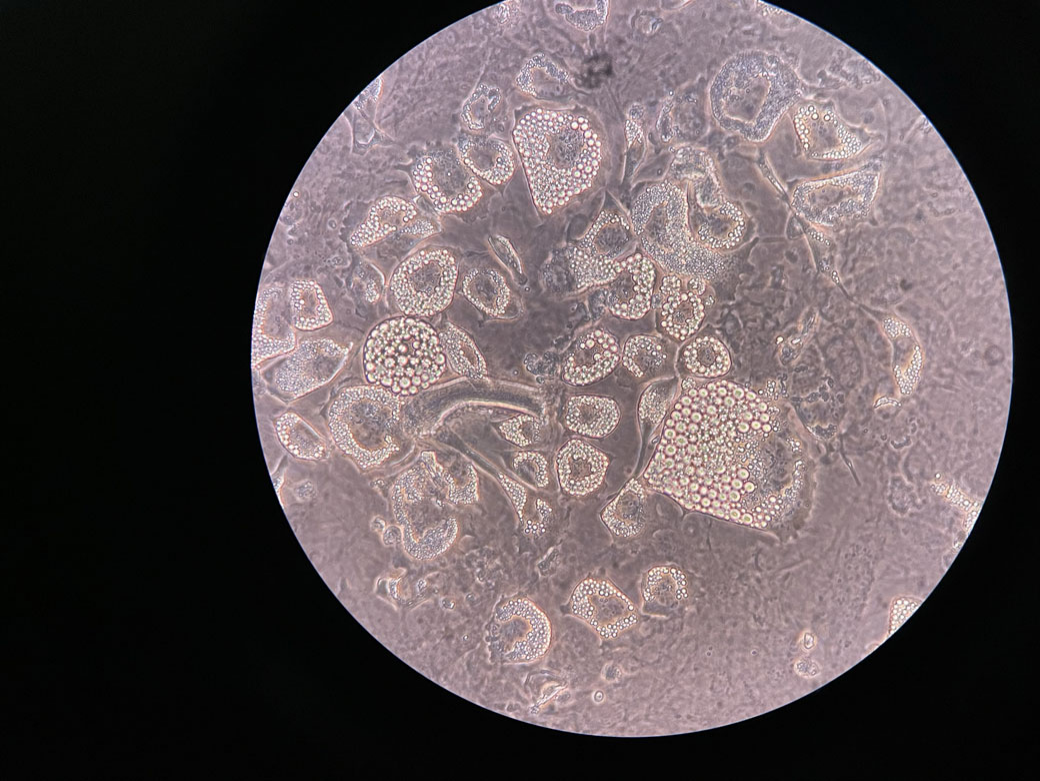
**When mouse ear-derived fibroblasts are grown in enriched media, lipid-like cells contaminate the monolayer.** Lipids appear in this photo as bright, spherical and organized between a cell membrane and a dense nucleus. These lipid-like cells contrast with the spindle and stellate-like mat of fibroblast cells below them. Image acquired on an iPhone camera with light microscopy at 40×.

**Which part of this research project was the most rewarding?**

I spent months troubleshooting the age, sex and genetic background of the cell donor mice, and even attempted to grow non-lipid cells after physically sorting out the unwanted lipid-like cells. The variable leading to the lipid-like cell contamination was much more mundane: the growth medium itself. The moment this realization and hypothesis stuck was the most rewarding, followed by the lesson I learned from this project: sometimes, the simplest answer is the right one, and right in front of you the whole time.

**What do you enjoy most about being an early-career researcher?**

I love learning. There is a freedom in being an early-career researcher that I deeply appreciate – exploring across fields, following unexpected threads and asking the simple questions.

**What piece of advice would you give to the next generation of researchers?**

Find your own perfect growth medium. Not every cell thrives under the same conditions, and neither does every scientist. The environment matters, so seek out conditions where you can grow. And don't mistake unculturable conditions for conditions we have not yet optimized (adapted from Montgomery, 2020).

**What's next for you?**

After defending my thesis, I am excited to bring my research and communications skills into the pharmaceutical industry through an internship with Abbott Diagnostics. Transitioning to research tools directly used to predict human health is an exciting next step in my career journey.

## References

[BIO062661C1] Barnes, K. L., Davis, N. M., Erazo, B. J., Cataldo, K. M., Bertges, E. H. and Knoll, L. J. (2026). Isolation, validation, and long-term culture of mouse ear fibroblasts. *Biol. Open* 15, bio062483. 10.1242/bio.062483 42046527 PMC13225709

[BIO062661C2] Montgomery, B. L. (2020). Lessons from microbes: what can we learn about equity from unculturable bacteria? *mSphere* 5, e01046-20. 10.1128/mSphere.01046-20 33115840 PMC7593602

